# Extraction, Purification, Structural Elucidation, and Antioxidant Assessment of the Bioactive Polysaccharides From Xinjiang Dried Apricot

**DOI:** 10.1155/jamc/2526753

**Published:** 2026-09-21

**Authors:** Hongying Ma, Xiaotian Wang, Zhongyang Li, Huakun Ying, Cheng Ma, Shuailian Zhao, Jiangli Lin, Bin Wu

**Affiliations:** ^1^ College of Chemistry, Xinjiang University, Urumqi 830046, China, xju.edu.cn; ^2^ School of Energy and Power, Xinjiang Engineering Vocational University, Tiemen Pass 841007, China; ^3^ School of Chemical Engineering and Technology, Xinjiang University, Urumqi 830046, China, xju.edu.cn; ^4^ School of Chemical Engineering, Xinjiang University, Urumqi 830046, China, xju.edu.cn; ^5^ Institute of Storage and Processing of Agricultural Products, Xinjiang Academy of Agricultural Sciences, Urumqi 830091, China, xaas.ac.cn/

**Keywords:** antioxidant activity, structural characterization, Xinjiang dried apricot of polysaccharides

## Abstract

**Background:**

Xinjiang apricots (*Prunus armeniaca* L.) are rich in bioactive polysaccharides with antioxidant, anti‐inflammatory, and immunomodulatory properties. As natural antioxidants, such polysaccharides offer advantages over synthetic counterparts in mitigating ROS‐related diseases. Their antioxidant depends on structural features, yet Xinjiang apricot polysaccharides remain poorly characterized. This study employs ultrasound‐assisted enzymatic extraction, DEAE‐52 purification, and multitechnique structural analysis to investigate these polysaccharides, aiming to establish their structure–activity relationship for potential functional food or therapeutic applications.

**Results:**

In this study, a galacturonic acid‐rich heteropolysaccharide (UEDAP‐4) was successfully extracted and isolated from dried apricots from Xinjiang, China. The polysaccharide is primarily composed of five monosaccharides: arabinose (Ara), rhamnose (Rha), galactose (Gal), glucose (Glc), and galacturonic acid (GalA), with a molar ratio of 28.78:20.36:6.87:5.51:38.48. The main chain of UEDAP‐4 is characterized by the following structure:⟶4)‐α‐D‐GalpA‐(1 ⟶ 5)‐α‐L‐Araf‐(1 ⟶ 4)‐α‐D‐GalpA‐(1 ⟶ 2)‐α‐L‐Araf‐(1 ⟶ 3)‐α‐L‐Araf‐(1 ⟶ 4)‐α‐D‐GalpA‐(1 ⟶ 2)‐α‐L‐Rhap‐(1 ⟶ 4)‐α‐L‐Rhap‐(1 ⟶ 4)‐α‐D‐GalpA‐(1⟶, with three additional branched chains. The half‐maximal inhibitory concentrations (IC_50_) of UEDAP‐4 against DPPH·, ·OH, and ABTS·^+^ were determined to be 5.399, 8.984, and 14.151 mg/mL, respectively. These results demonstrate that UEDAP‐4 exhibits potent free radical scavenging activity, likely due to its high content of GalA and Ara.

**Conclusions:**

This study significantly advances our understanding of the relationship between the structural properties of polysaccharides derived from Xinjiang dried apricots and their antioxidant activity. This discovery highlights UEDAP‐4 as a promising candidate for natural antioxidants, with potential applications in food preservation, pharmaceutical formulations, and functional food development.

## 1. Introduction

The apricot tree (*Prunus armeniaca* L.) is a member of the *Prunus* genus [1] and is extensively cultivated in Xinjiang, China, ranking as the third largest fruit tree globally in terms of planted area [2]. China is the world’s leading producer of apricots. Xinjiang, its primary growing region, alone had a planting area of 116,300 ha and an annual production of 937,600 tons in 2020 [3]. The unique geographical location of Xinjiang results in a dry climate, low precipitation, and prolonged sunshine duration. Consequently, apricots from this region exhibit high sugar content and are rich in nutrients and bioactive compounds, which are beneficial for human health [4]. Xinjiang apricots are characterized by thin skin, high juiciness, and high perishability. As a result, more than 95% of fresh apricots in Xinjiang are processed into dried apricots [5]. Dried apricots are rich in biologically active polysaccharides, which are composed of multiple monosaccharide units linked by glycosidic bonds to form polymerized carbohydrate structures. These polysaccharides demonstrate significant anti‐inflammatory, antitumor, and antioxidant properties, as well as the ability to reduce blood lipid levels, regulate blood glucose levels, and enhance immune responses [6–9]. Therefore, dried apricots with high polysaccharide content possess significant food and medicinal value.

Studying the antioxidant activity of polysaccharides derived from dried apricots is critical for reducing the risk of disease development. Common reactive oxygen species (ROS) include hydroxyl radicals (−OH), superoxide anions (O_2_ · ^−^), and hydrogen peroxide (H_2_O_2_) [10]. Excessive levels of ROS can induce oxidative stress, which may contribute to the development of cardiovascular diseases (CVD), atherosclerosis, and diabetes [11]. Although some synthetic antioxidants can inhibit the production of excessive ROS, they are often associated with adverse side effects and potential health risks [12]. In contrast, natural antioxidants are considered more reliable and accessible, exhibiting anti‐inflammatory, anticancer, cardioprotective, neuroprotective, and antiaging properties. Therefore, the exploration and extraction of natural antioxidants are of significant importance.

Research has demonstrated that polysaccharides derived from natural products can effectively scavenge excessive ROS in vivo, thereby exhibiting potent antioxidant activity [13–15]. Furthermore, it has been established that specific structural features of polysaccharides, including molecular weight (*M*_*W*_), monosaccharide composition, glycosidic bond configuration, and linkage types, are closely associated with their antioxidant properties [16]. Therefore, the isolation, purification, and structural characterization of polysaccharides from diverse natural sources are crucial for elucidating the relationship between polysaccharide structure and antioxidant activity. This knowledge provides a foundation for future research into the structure–activity relationships of bioactive polysaccharides and the development of natural antioxidants for practical applications. Zhang et al. [16] successfully extracted, isolated, and purified four polysaccharide fractions (PKP‐E‐1‐1, ‐1‐2, ‐2‐1, and ‐2‐2) from the pinecones of *Pinus koraiensis*. Among these fractions, PKP‐E‐2‐1 exhibits a relatively low molecular weight and a high content of L‐rhamnose, L‐arabinose, and D‐galactose. This composition contributes to its potent scavenging activity against hydroxyl radicals and ABTS^+^ radicals, indicating significant antioxidant potential. Ji et al. [17] successfully extracted, isolated, and purified four polysaccharide fractions (GZMP‐1, GZMP‐2, GZMP‐3, and GZMP‐4) from *Ziziphus Jujuba* cv. Muzao. GZMP‐4 demonstrated superior scavenging capacity for both DPPH and hydroxyl radicals, attributed to its high galacturonic acid content. This indicates that GZMP‐4 may have the potential as a potent antioxidant. Wu et al. [18] found that among the three polysaccharide fractions (PHP1, PHP2, and PHP3) extracted, isolated, and purified from *Porphyra haitanensis*, PHP3 displayed the highest scavenging activity against DPPH and hydroxyl radicals, owing to its high sulfate content. In contrast, PHP1, with the lowest sulfate content, exhibited significantly weaker scavenging activity against DPPH and hydroxyl radicals.

Currently, there is limited research on the purification and structural characterization of polysaccharides derived from Xinjiang dried apricots in the academic literature. Investigating Xinjiang dried apricot polysaccharides enhances our understanding of their structure–activity relationships and provides valuable insights for the development of natural antioxidants from polysaccharides. In this study, polysaccharides were extracted from Xinjiang dried apricots using an ultrasound‐assisted enzymatic method. The polysaccharides were subsequently isolated and purified using cellulose DEAE‐52 anion‐exchange column chromatography. The antioxidant activities of the purified polysaccharides were then evaluated. The structural characteristics of the polysaccharides were characterized using high‐performance anion‐exchange chromatography (HPAEC), gas chromatography–mass spectrometry (GC–MS), Fourier transform infrared (FTIR) spectroscopy, and nuclear magnetic resonance (NMR) spectroscopy.

## 2. Materials and Methods

### 2.1. Materials and Reagents

Dried apricots (var. Diaoganxing) sourced from the Fourth Regiment of Aksu City, Xinjiang, were purchased from the Urumqi Dried Fruit Market (Urumqi, China). DEAE‐52 cellulose and 2,2‐Alanylbis (3‐ethylbenzothiazoline‐6‐sulfonate) (ABTS) were obtained from Beijing InnoChem Science & Technology Co., Ltd. (Beijing, China). 2,2‐Diphenyl‐1‐picrylhydrazyl (DPPH) and Sephadex G‐200 were purchased from Yishijiu Biotechnology Co., Ltd. (Lianyungang, Jiangsu, China). Cellulase (98%) was obtained from Shanghai McLean Biochemical Technology Co., Ltd. (Shanghai, China), and pectinase (food grade) was sourced from Xi’an Yuhua Biotechnology Co. (Xi’an, Shanxi, China). Methanol was purchased from ANPEL Laboratory Technologies (Shanghai, China), while acetic acid, methylene chloride, and trifluoroacetic acid (TFA) were obtained from CNW Technologies GmbH (Düsseldorf, Germany). Dimethyl sulfoxide (DMSO), sodium borodeuteride, acetic anhydride, sodium hydroxide, sodium acetate trihydrate, rhamnose (Rha), galactose (Gal), galacturonic acid (GalA), xylose (Xyl), arabinose (Ara), mannose (Man), fucose (Fuc), mannuronic acid (ManA), fructose (Fru), glucose (Glc), ribose (Rib), guluronic acid (GulA), and glucuronic acid (GlcA) were purchased from Sigma‐Aldrich (St. Louis, MO, USA). All other chemical reagents, which were of analytical grade, were obtained from Tianjin Zhiyuan Chemical Reagent Co., Ltd. (Tianjin, China).

### 2.2. Pretreatment of Dried Apricot Samples

The apricots were pitted, chopped, dried to constant weight, crushed, and sieved. The resulting powder was first treated with petroleum ether (1:6, w/v) at 65°C for 2 h and then with 80% ethanol (1:6, w/v) under the following conditions: soaking for 3 h, followed by refluxing at 80°C for 1 h. After each solvent treatment, the mixture was centrifuged. The final precipitate was washed twice with 95% ethanol and dried to constant weight at 50°C in a vacuum drying oven, yielding defatted apricot powder (yield: 98.65 ± 0.12%).

### 2.3. Extraction of the Dried Apricot Polysaccharides and Single‐Factor Experiments

In a 100 mL beaker, 2.0 g of defatted apricot powder was mixed with an enzyme complex (cellulase: pectinase) at specific concentrations (0.5%–2.5%) and ratios (1:1–3:1). Aqueous solutions with varying pH levels (4.0–6.0) were added at different liquid‐to‐solid ratios (20:1–40:1 mL/g). Polysaccharides were extracted via ultrasonication at varying temperatures (30°C–70°C) and extraction durations (15–55 min). Following extraction, the mixture was boiled to inactivate the enzymes. After centrifugation, the supernatant was collected and concentrated. Proteins were removed using the trichloroacetic acid precipitation method [19]. Dried apricot crude polysaccharide (UEDAP) was obtained by adding anhydrous ethanol (1:4, v/v) to the extract, followed by alcohol sedimentation at 4°C for a duration of 24 h to collect the precipitate. The polysaccharide yield (%) was calculated using the following formula:

(1) Y%=M1M0×100%,

where *Y* is the extraction yield of polysaccharides (%), *M*_1_ is the mass of dried polysaccharides (g), and *M*_0_ is the mass of defatted apricot powder (g).

### 2.4. Experimental Design of RSM

#### 2.4.1. Optimization of UEDAP by RSM

To optimize the extraction conditions of UEDAP, the Box–Behnken design (BBD) with three independent variables was employed, based on preliminary single‐factor experiments. Among the six factors examined, three variables were identified as having the most significant impact on UEDAP yield: enzyme ratio (A), enzyme concentration (B), and pH (C) [20]. The enzyme ratio was selected because the synergistic action of cellulase and pectinase is critical for degrading cell wall components [21]. Enzyme concentration directly governs the extent of enzymatic hydrolysis; insufficient concentration leads to incomplete cell wall disruption, while excessive concentration may cause polysaccharide degradation [22]. pH was chosen due to its strong influence on enzyme activity [23]. The variable ranges were defined as follows: enzyme ratio (1.5:1–2.5:1), enzyme concentration (1.5%–2.5%), and pH (5.0–6.0), ensuring that the optimal conditions were encompassed within the experimental domain [20]. Each independent variable was coded at three levels (−1, 0, +1), with extraction yield (Y) as the response variable. The factors and their corresponding levels are presented in Table 1. The experimental design comprised 17 randomized runs, as detailed in Table 2.

**TABLE 1:** Box–Behnken test level table.

Level	A: Ratio of complex enzymes	B: Concentration of complex enzymes (%)	C: pH
−1	1.5	1.5	5.0
0	2.0	2.0	5.5
1	2.5	2.5	6.0

**TABLE 2:** Box–Behnken design matrix (in the coded level of three variables) and response values for the yield of UEDAP.

Number	Factors			Extraction yield (%)
	A: Ratio of complex enzymes	B: Concentration of complex enzymes (%)	C: pH	
1	1	−1	0	24.36
2	0	0	0	34.40
3	0	0	0	36.73
4	0	0	0	34.73
5	0	−1	−1	19.30
6	0	0	0	35.79
7	0	−1	1	26.04
8	0	1	−1	30.54
9	0	1	1	30.93
10	1	0	1	30.38
11	1	0	−1	27.51
12	−1	0	−1	26.24
13	−1	0	1	29.01
14	1	1	0	28.8
15	−1	−1	0	21.29
16	0	0	0	33.85
17	−1	1	0	28.52

#### 2.4.2. Verification of the Predicted Optimized Conditions

To validate the developed mathematical model, experiments were conducted in triplicate under the optimized conditions. The accuracy and validity of the optimized conditions were confirmed by comparing the mean experimental value with the predicted value derived from the established model.

### 2.5. Isolation and Purification of UEDAP

The decolorized dried apricot polysaccharide (25 mg) was dissolved in ultrapure water (5 mg/mL) and loaded onto a DEAE‐52 cellulose column (2.5 × 75 cm). Gradient elution was carried out sequentially using NaCl solutions (0, 0.05, 0.1, 0.15, 0.2, 0.3, 0.4, and 0.5 M) at a flow rate of 1 mL/min. The polysaccharide content in the eluate was measured using the phenol–sulfuric acid method, with 10 mL fractions collected per tube. The major fractions were pooled, concentrated, dialyzed, and freeze‐dried to obtain the purified dried apricot polysaccharide (UEDAP‐4). UEDAP‐4 was further purified and analyzed using Sephadex G‐200 (60 × 1 cm) gel chromatography. The chromatographic conditions were as follows: A 0.3 M sodium chloride solution was used as the mobile phase at a flow rate of 0.4 mL/min. A sample volume of 20 μL was injected, and the column temperature was maintained at 25°C. The signal was detected using a differential refractive index detector.

### 2.6. Structural Analysis of UEDAP‐4

#### 2.6.1. UV and FTIR Analysis

A UV/Vis spectrophotometer (UV‐2600, Shimadzu, Kyoto, Japan) was used to record the UV spectrum over a scanning range of 200–800 nm, allowing for the detection of proteins and nucleic acids in the polysaccharides.

For FTIR analysis, UEDAP‐4 was mixed with KBr, pressed into pellets, and analyzed using a FTIR spectrometer over a wavenumber range of 4000 to 500 cm^−1^.

#### 2.6.2. Analysis of Monosaccharides

The UEDAP‐4 compound was analyzed by HPAEC [24]. UEDAP‐4 (5 mg) was hydrolyzed by adding 1 mL of 2 M TFA and heating at 121°C for 2 h. Nitrogen blow‐drying was performed. Methanol (1 mL) was added to the hydrolysate for dissolution, and this step was repeated at least three times. The solution was then dried under a nitrogen stream to remove residual TFA, redissolved in deionized water, and filtered through a 0.22 μm membrane to obtain the final solution for analysis. The monosaccharide composition of UEDAP‐4 was analyzed using a pulsed amperometric detector (PAD) coupled with a Thermo ICS 5000+ ion chromatography system. A Dionex CarboPac PA20 column (150 × 3.0 mm, 10 μm) was used at a flow rate of 0.5 mL/min with an injection volume of 5 μL. The mobile phases consisted of (A) H_2_O, (B) 0.1 M NaOH, and (C) 0.1 M NaOH with 0.2 M NaAc, delivered at a flow rate of 0.5 mL/min, with the column temperature maintained at 30°C. Mixed monosaccharide standards were pretreated and analyzed following the same procedure as for UEDAP‐4. The monosaccharide composition of UEDAP‐4 was identified based on retention times, and quantification was performed using external standard methods.

#### 2.6.3. Analysis by Scanning Electron Microscope (SEM)

The molecular morphology of polysaccharides was observed using a benchtop high‐field microscope (TM3030, Hitachi High‐Technologies Co., Tokyo, Japan). The polysaccharide samples were adhered to a sample holder using double‐sided tape. After applying gold sputtering to the samples, their surface structures were examined and captured on the SEM at magnifications of 100x and 500x.

#### 2.6.4. Methylation Analysis

UEDAP‐4 was methylated following a modified Hakomori method [25]. Prior to methylation, UEDAP‐4 was reduced with NaBD_4_, converting the uronic acid residues into their corresponding neutral sugars. The reduced UEDAP‐4 was dissolved in anhydrous DMSO and then methylated with CH_3_I in a mixture of DMSO and NaOH. Following methylation, the sample was hydrolyzed with 2 M TFA at 121°C for 1.5 h. Subsequently, it was reduced with NaBD_4_ and acetylated with acetic anhydride at 100°C for 2.5 h. The partially methylated alditol acetates (PMAAs) were analyzed by GC–MS (Agilent 6890A‐5977B) equipped with an Agilent BPX70 column (30 m × 0.25 mm × 0.25 μm), using helium as the carrier gas at a split ratio of 10:1. A 1 μL sample was injected for GC–MS analysis. The temperature program began at 140°C for 2.0 min, followed by a ramp to 230°C at a rate of 3°C/min, and held at 230°C for an additional 3 min. The scan mode was set to full scan, with a mass‐to‐charge ratio (m/z) range of 50–350.

#### 2.6.5. NMR Analysis

The UEDAP‐4 solution was prepared using D_2_O at a concentration of 50 mg/mL. The solution was analyzed using an NMR spectrometer to acquire ^1^H NMR, ^13^C NMR, ^1^H‐^1^H COSY, ^1^H‐^13^C HSQC, ^1^H‐^13^C HMBC, ^1^H‐^1^H NOESY, and DEPT 135 spectra. The NMR spectra were acquired on a Bruker Avance III 600 MHz spectrometer at a probe temperature of 323.15 K [26].

### 2.7. Antioxidant Activity Assay

#### 2.7.1. DPPH Radical Scavenging Assay

The DPPH radical scavenging activity of UEDAP‐4 was determined following the method described by Fan et al. [27]. Polysaccharide solutions (1.0 mL) at varying concentrations (0.0, 0.5, 1.5, 2.5, and 3.5 mg/mL) were mixed with 1.0 mL of 0.025 mM DPPH in 95% ethanol. The mixtures were incubated in the dark for 30 min, and the absorbance was measured at 519 nm. Ascorbic acid (Vitamin C, Vc) was used as a positive control. The DPPH radical scavenging activity (%) was calculated using the following formula:

(2) DPPH radical scavenging activity %=1−A1−A2A0×100 %,

where *A*_1_ is the absorbance of the sample, *A*_2_ is the absorbance of the sample without DPPH, and *A*_0_ is the absorbance of the blank (water instead of the sample).

#### 2.7.2. Hydroxyl Radical Scavenging Assay

The hydroxyl radical scavenging activity of the polysaccharides was determined using the salicylic acid method, as described by Chen et al. [28]. Polysaccharide solutions (UEDAPs) at varying concentrations (1 mL; 1, 2, 3, 4, and 5 mg/mL) were mixed with 1.0 mL of 9 mM ferrous sulfate, 1.0 mL of 9 mM salicylic acid (in absolute ethanol), and 1.0 mL of 8.8 mM H_2_O_2_, followed by incubation at 37°C for 30 min. The absorbance was measured at 530 nm. Vc was used as a positive control. The hydroxyl radical scavenging activity (%) was calculated using the following formula:

(3) Hydroxyl radical scavenging activity %=1−A1−A2A0×100 %,

where *A*_1_ is the absorbance of the sample, *A*_2_ is the absorbance of the sample without hydroxyl radicals, and *A*_0_ is the absorbance of the blank (water instead of the sample).

#### 2.7.3. ABTS^⋅+^ Radical Scavenging Assay

The ABTS^⋅+^ radical scavenging activity of UEDAPs was determined following the method described by Xie et al. [29]. The ABTS^⋅+^ stock solution was diluted to achieve an absorbance of 0.70 ± 0.02 at 734 nm. Polysaccharide solutions (0.3 mL) at varying concentrations were mixed with 3.0 mL of diluted ABTS solution and incubated at room temperature for 6 min. The absorbance of the mixture was measured at 734 nm. Vc was used as a positive control. The ABTS^⋅+^ radical scavenging activity (%) was calculated using the following equation:

(4) ABTS·+radical scavenging activity %=1−A1−A2A0×100 %,

where *A*_1_ is the absorbance of the sample, *A*_2_ is the absorbance of the sample without ABTS^⋅+^ radicals, and *A*_0_ is the absorbance of the blank (water instead of the sample).

### 2.8. Statistical Analysis

All measurements were performed in triplicate, and the data are presented as mean ± standard deviation (SD). Statistical analyses were conducted using GraphPad Prism 8.0 (GraphPad Software, Inc., San Diego, CA, USA). One‐way analysis of variance (ANOVA) was used to evaluate differences between groups, with *p* < 0.05 considered statistically significant.

## 3. Results and Discussion

### 3.1. Effect of Extraction Conditions on the Extraction Yield of UEDAP

**FIGURE 1:**
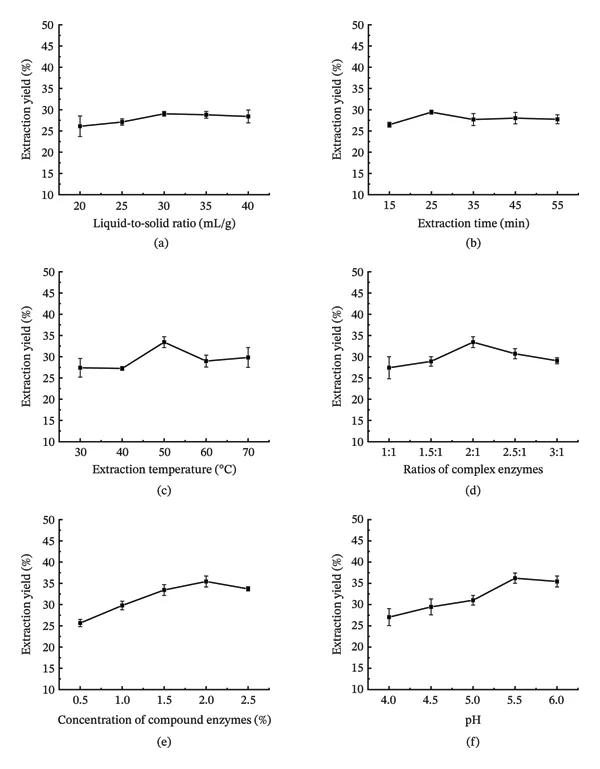
Effects of different extraction parameters on the extraction yield of UEDAP: (a) the liquid‐to‐solid ratios, (b) extraction time, (c) extraction temperature, (d) ratio of compound enzymes, (e) concentration of compound enzymes, and (f) pH.

*Liquid-to-solid ratios*: The effect of liquid‐to‐solid ratios on the extraction yield of UEDAP is shown in Figure 1a. As the liquid to solid ratio increased from 20 to 40 mL/g, the extraction yield of UEDAP initially increased and then decreased. Notably, the UEDAP extraction yield reached a maximum of 29.05 ± 0.01 g per 100 g of dried apricot powder (29.05 ± 0.01% w/w) at a liquid‐to‐solid ratio of 30 mL/g. A higher solvent volume increases the concentration gradient between the intracellular and extracellular environments, promoting polysaccharide diffusion into the solvent and enhancing the extraction yield. However, excessive solvent may dissolve impurities from dried apricots, reducing the polysaccharide concentration in the solution and ultimately decreasing the extraction yield [30]. Therefore, a liquid‐to‐solid ratio of 30 mL/g was selected as the optimal condition for further studies.

*Extraction time*: The effect of extraction time on the extraction yield of UEDAP is shown in Figure 1b. The extraction yield of UEDAP initially increased, then decreased with prolonged extraction time, and eventually stabilized. The maximum extraction yield of 29.45 ± 0.45 g per 100 g of dried apricot powder (29.43 ± 0.45% w/w) was achieved at an extraction time of 25 min. This trend may be explained by progressive damage to plant cells as the extraction time increases. This damage enhances polysaccharide diffusion into the solvent, thereby increasing the UEDAP extraction yield. However, prolonged extraction may lead to polysaccharide degradation, resulting in a gradual decline and stabilization of the extraction yield [31]. Therefore, the optimal extraction time was determined to be 25 min.

*Extraction temperature*: The effect of extraction temperature on the extraction yield of UEDAP is shown in Figure 1c. The extraction yield of UEDAP initially increased and then decreased with increasing extraction temperature. The maximum extraction yield of UEDAP, reaching 33.43 ± 1.27 g per 100 g of dried apricot powder (33.43 ± 1.27% w/w), was achieved at an extraction temperature of 50°C. The solubility of polysaccharides increased with temperature due to enhanced molecular motion, which promoted UEDAP solubilization. However, when the extraction temperature exceeded 50°C, the thermal effect caused degradation of the polysaccharide structure. This resulted in faster degradation of the polysaccharide structure relative to its dissolution, leading to a significant reduction in extraction yield [32]. Therefore, an extraction temperature of 50°C was selected as optimal for further studies.

*Ratio of compound enzymes*: The effect of the ratio of compound enzymes on the extraction yield of UEDAP is shown in Figure 1d. It was observed that the extraction yield of UEDAP initially increased and then decreased with an increase in the ratio of compound enzymes. The maximum extraction yield of UEDAP, reaching 33.43 ± 1.27 g per 100 g of dried apricot powder (33.43 ± 1.27% w/w), was achieved at a ratio of compound enzymes of 2:1. Studies have indicated that the plant cell wall is primarily composed of cellulose, hemicellulose, and pectin. Cellulose is a key structural component of the primary cell wall, while pectin is a major constituent of the secondary cell wall [33]. The cellulase content increased correspondingly with the increase in the ratio of the composite enzyme (cellulase and pectinase). These enzymes enhance the solubility of the primary cell wall, promoting the diffusion of polysaccharides. Consequently, this process increases the polysaccharide extraction yield. At higher compound enzyme ratios, the reduced pectinase concentration decreases the disruption of the secondary cell wall. This ultimately reduces the polysaccharide extraction yield [34]. Therefore, the optimal ratio of compound enzymes was selected as 2:1 for further studies.

*Concentration of compound enzymes*: The effect of the concentration of compound enzymes on the extraction yield of UEDAP is shown in Figure 1e. It was observed that the extraction yield of UEDAP initially increased and then decreased with an increase in the concentration of compound enzymes. The maximum extraction yield of UEDAP, reaching 35.43 ± 1.29 g per 100 g of dried apricot powder (35.43 ± 1.29% w/w), was achieved at a concentration of 2.0% for compound enzymes. This phenomenon occurs because increasing the compound enzyme concentration enhances the activity of cellulase and pectinase. This enhances cell wall hydrolysis by the compound enzyme, reducing the mass transfer resistance of polysaccharides. Consequently, the diffusion coefficient increases, accelerating polysaccharide dissolution. However, when the polysaccharide becomes saturated, a high concentration of the complex enzyme may lead to hydrolysis of glycosidic bonds and consequently reduce the yield of polysaccharide extraction [35]. Therefore, the optimal concentration of compound enzymes was determined to be 2.0% for further studies.

*pH*: The effect of pH on the extraction yield of UEDAP is shown in Figure 1f. It can be observed that the extraction yield of UEDAP initially increases and then decreases with an increase in pH, reaching a peak value of 36.22 ± 1.22 g per 100 g of dried apricot powder (36.22 ± 1.22% w/w) at pH 5.5. pH significantly affects enzyme activity. As pH increases, the activity of the compound enzyme and the solubility of polysaccharides increase [36]. However, enzyme activity reaches its optimum within a specific pH range. Beyond this range, further increases in pH reduce compound enzyme activity, resulting in a decline in polysaccharide extraction yield. Therefore, a pH of 5.5 was determined to be optimal based on the experimental results.

### 3.2. Optimization of UEDAP Extraction Conditions by RSM

#### 3.2.1. Statistical Analysis and Model Fitting

A total of 17 experiments were performed to optimize three independent variables. The relationship between the response and independent variables was described by a second‐order polynomial equation derived from multiple regression analysis of the experimental data. The equation is as follows:

(5) Y=+35.100.753.481.600.700.031.593.895.472.93+∗A+∗B+∗C−∗AB+∗AC−∗BC−∗A2−∗B2−∗C2,

where *A*, *B*, and *C* represent the ratios of compound enzymes, concentrations of compound enzymes, and pH, respectively. *Y* denotes the predicted yield of UEDAP.

The ANOVA results for the response surface quadratic model are presented in Table 3. The model was highly significant, as indicated by a low *p* value (*p* < 0.0001) and a high *F*‐value of 36.59. Additionally, the lack of fit was not statistically significant (*p* > 0.05), further validating the model’s accuracy. The regression coefficient *R* ^2^ = 0.9792 indicates that the equation is well‐fitted and that it can be used to replace the test true point to describe the relationship between the variables and the response values. The *R* ^2^ value of 0.9792 and the $R_{\text{adj}}^{2}$ value of 0.9524, both being close to 1, suggest a strong correlation between the experimental and predicted values, indicating the model’s high degree of accuracy. Therefore, the model is suitable for analyzing and predicting the effects of various factors on the extraction yield during the ultrasound‐assisted enzymatic extraction of UEDAP.

**TABLE 3:** Analysis of variance for the fitted regression model equation.

Source	Sum of squares	DF mean	Mean square	*F*‐value	*p*‐value	Significance^∗^
Model	383.43	9	42.60	36.59	< 0.0001	^∗∗^
A	4.49	1	4.49	3.85	0.0905	
B	96.61	1	96.61	82.98	< 0.0001	^∗∗^
C	20.38	1	20.38	17.51	0.0041	^∗∗^
AB	1.95	1	1.95	1.67	0.2371	
AC	0.0025	1	0.0025	0.0021	0.9643	
BC	10.08	1	10.08	8.66	0.0216	^∗^
A^2^	63.63	1	63.63	54.66	0.0002	^∗∗^
B^2^	125.98	1	125.98	108.21	< 0.0001	^∗∗^
C^2^	36.09	1	36.09	31.00	0.0008	^∗∗^
Residual	8.15	7	1.16			
Lack of fit	2.83	3	0.9423	0.7082	0.5955	
Pure error	5.32	4	1.33			
Cor total	391.58	16				
	*R* ^2^ = 0.9792		$R_{\text{Adj}}^{2}$ = 0.9524			

*Note:* ^∗^< 0.05 significant differences, ^∗∗^< 0.01 very significant differences.

From the *p* values in Table 3, it can be seen that in the primary term, the effect of the ratio of compound enzymes (A) on the extraction yield is not significant, the effect of the concentration of compound enzyme (B) is highly significant, and the effect of pH (C) is highly significant in the secondary term, and the effects of A^2^, B^2^, and C^2^ on the extraction yield are highly significant. In the interaction term, AB and AC had no significant effects on the extraction yield, whereas BC showed a significant effect. Based on the *F*‐values in Table 3, the influence of the three factors on the extraction yield of UEDAP followed the order: the concentration of compound enzymes (B) > pH (C) > ratio of compound enzymes (A).

#### 3.2.2. Analysis of Response Surfaces

**FIGURE 2:**
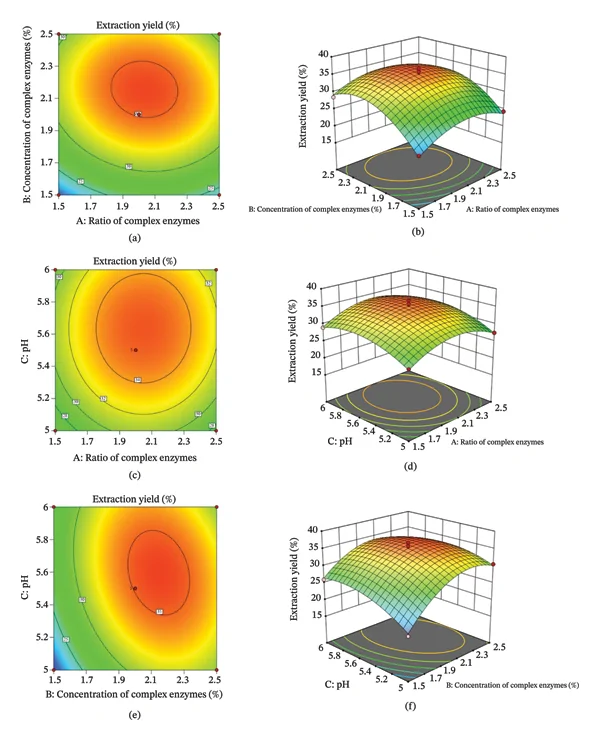
Effects of ratio of compound enzymes and concentration of compound enzymes on the extraction yield of UEDAP (a, b), effects of ratio of compound enzymes and pH on the extraction yield of UEDAP (c, d), and effects of concentration of compound enzymes and pH on the extraction yield of UEDAP (e, f).

To explore the interaction between variables and ascertain the best levels for achieving the maximum response, 3D response surfaces and 2D contour plots were created as depicted in Figure 2. A circular contour plot indicates minimal interaction between the variables, while an elliptical contour plot indicates significant interaction [37]. Figure 2a,b illustrates the interactions between the compound enzyme ratio and concentration on the UEDAP yield at pH 5.5. It was observed that as the ratio of compound enzymes increased, there was minimal change in the extraction yield of UEDAP. However, with an increase in the concentration of compound enzymes, the extraction yield of UEDAP initially increased and then decreased. The interaction of the ratio of compound enzymes and concentration of compound enzymes was not significant. The UEDAP yield affected by different ratios of compound enzymes and pH was shown in Figure 2c,d, with the concentration of compound enzymes fixed at a zero level (2.0%). The yield rate of UEDAP increased at first and then decreased with the increasing pH. The interaction of the ratio of compound enzymes and pH was not significant. The UEDAP yield affected by different concentrations of compound enzymes and pH was shown in Figure 2e,f, with the ratio of compound enzymes fixed at a zero level (2:1). The UEDAP yield initially increased and then decreased with increasing enzyme concentration and pH. The interaction between the two factors of concentration of compound enzymes and pH was significant (*p* < 0.05).

#### 3.2.3. Verification of the Predictive Model

The optimal ultrasound‐assisted enzymatic extraction conditions for UEDAP extraction given by Design‐Expert software were as follows: The enzyme complex ratio was 2.04, the concentration of compound enzymes was 2.14%, and the pH was 5.60. At the optimal conditions, the predicted maximum yield of UEDAP reached 35.78 g per 100 g of dried apricot powder (35.78% w/w). Considering the reliability of the experimental process, the following were identified as the optimal extraction conditions to achieve the highest yield of polysaccharides: The enzyme complex ratio was 2.00, the concentration of compound enzymes was 2.00%, and the pH was 5.50. Under these conditions, the experiment yield of UEDAP was 35.53 ± 0.60 g per 100 g of dried apricot powder (35.53 ± 0.60% w/w). This result closely matches the model’s predicted value, indicating a good fit between the equation and experimental outcomes and demonstrating the response surface model’s effectiveness in predicting UEDAP extraction yields.

### 3.3. Isolation and Purification of UEDAP

**FIGURE 3:**
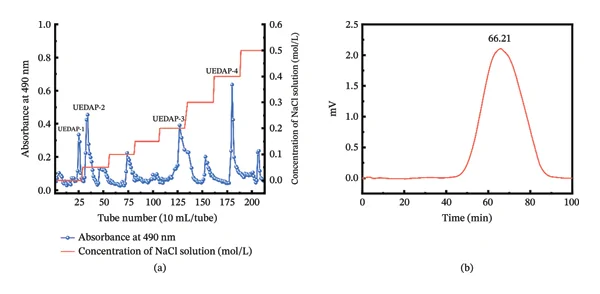
(a) Using a 0–0.5 M NaCl stepwise elution curve of UEDAP on a DEAE‐cellulose DE‐52 column, (b) Sephadex G‐200 gel chromatogram of UEDAP‐4.

The gradient elution profile of UEDAP on a DEAE‐cellulose DE‐52 anion exchange column is shown in Figure 3a. As shown in the figure, the chromatographic separation of UEDAP on the DEAE‐cellulose DE‐52 column yielded four polysaccharide fractions. Among these, the eluted fraction of 0.40 moL/L NaCl was identified as the major fraction, denoted as UEDAP‐4. UEDAP‐4 was further analyzed by Sephadex G‐200 gel chromatography, as shown in Figure 3b, which produced a single symmetric peak, confirming the homogeneity of UEDAP‐4.

### 3.4. Structural Analysis of UEDAP‐4

#### 3.4.1. UV and FTIR Analysis

**FIGURE 4:**
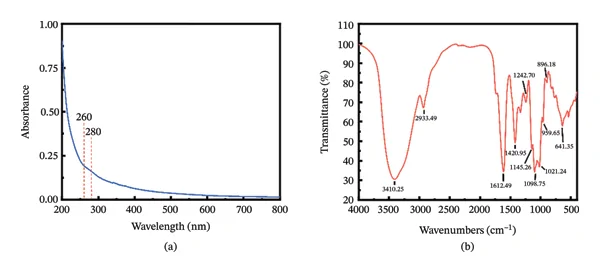
(a) UV spectrum of UEDAP‐4 and (b) FTIR spectrum of UEDAP‐4.

The UV spectrum of UEDAP‐4 (Figure 4a) showed no distinct absorption peaks at 260 nm or 280 nm within the range of 200–800 nm, indicating the absence of nucleic acids and proteins.

FTIR spectroscopy was used to analyze the functional groups and glycosidic linkages in UEDAP‐4. As shown in Figure 4b, the FTIR spectrum of UEDAP‐4 displayed a broad and intense absorption band at 3410.25 cm^−1^, primarily attributed to O‐H stretching vibrations, and a weaker band at 2933.49 cm^−1^, corresponding to C‐H stretching vibrations [38]. The absorption bands at 1612.49 and 1420.95 cm^−1^ were assigned to the asymmetric and symmetric stretching vibrations of the C=O bond in uronic acid, respectively [39]. Additionally, the peaks at 1145.26, 1098.75, and 1021.24 cm^−1^ confirmed the presence of C‐O‐C and C‐O‐H stretching vibrations, characteristic of the pyranose ring structure [40]. The absorption peaks at 959.65 and 896.18 cm^−1^ were attributed to the β‐configuration of the pyranose ring [41].

#### 3.4.2. Monosaccharide Fraction Analysis of UEDAP‐4

**FIGURE 5:**
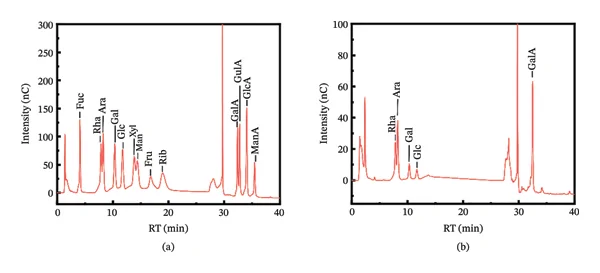
(a) Ion chromatography (ICS) profiles of monosaccharide standards and (b) monosaccharide composition of UEDAP‐4.

The monosaccharide composition of UEDAP‐4 was determined using ion chromatography. The monosaccharide composition of UEDAP‐4 was determined by combining the results with the ion chromatogram of the standard product (Figure 5a), as shown in Figure 5b. The results revealed that UEDAP‐4 is an acidic heteropolysaccharide composed of Ara, Rha, Gal, Glc, and GalA in a molar ratio of 28.78:20.36:6.87:5.51:38.48.

#### 3.4.3. SEM Analysis

**FIGURE 6:**
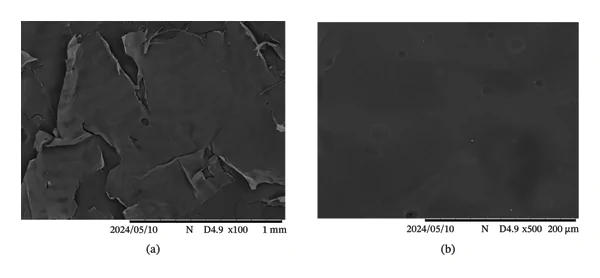
Scanning electron micrographs of UEDAP‐4 at magnification of 100x (a) and 500x (b).

SEM images of UEDAP‐4 at magnifications of 100x (Figure 6a) and 500x (Figure 6b) were used to analyze the surface morphology of the polysaccharide. At 100x magnification (Figure 6a), UEDAP‐4 exhibited an irregular sheet‐like structure with uneven surface dimensions. At 500x magnification (Figure 6b), UEDAP‐4 displayed a smooth and dense surface texture [42].

#### 3.4.4. Methylation Analysis of UEDAP‐4

**FIGURE 7:**
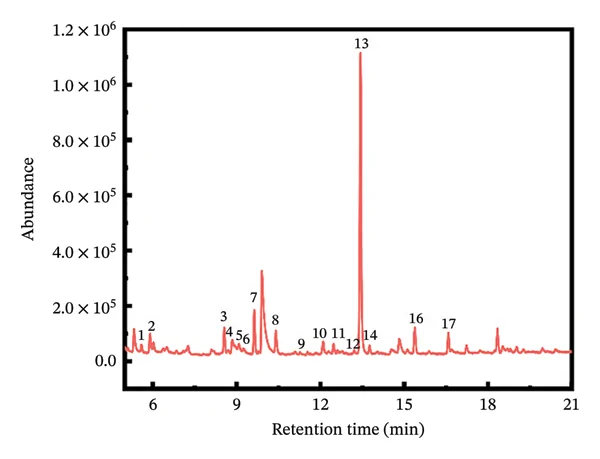
GC–MS total ion flow diagrams of PMAAs belonging to UEDAP‐4 (1‐17 were 2,3,4‐Me_3_‐Rhap, 2,3,5‐Me_3_‐Araf, 3,4‐Me_2_‐Rhap, 2,4‐Me_2_‐Rhap, 2,3‐Me_2_‐Rhap, 2,5‐Me_2_‐Araf, 2,3,4,6‐Me_4_‐GalpA, 2,3‐Me_2_‐Araf, 4‐Me‐Rhap, 3‐Me‐Rhap, 2,4,6‐Me_3_‐Galp, 2,3,6‐Me_3_‐Galp, 2,3,6‐Me_3_‐GalpA, 2,3,6‐Me_3_‐Glcp, 3‐Me‐Araf, 2,6‐Me_2_‐Galp, and 3,6‐Me_2_‐Glcp).

After undergoing methylation, hydrolysis, and acetylation, the types of glycosidic bonds present in purified dried apricot polysaccharides were determined by analyzing UEDAP‐4 using GC–MS and comparing the results with a standard mass spectral library. The results of the study are presented in Table 4. The GC–MS total ion chromatogram of PMAAs derived from UEDAP‐4 is shown in Figure 7. Consistent with the monosaccharide composition analysis, UEDAP‐4 consisted of Rhap‐(1⟶, Araf‐(1⟶, ⟶2)‐Rhap‐(1⟶, ⟶3)‐Rhap‐(1⟶, ⟶4)‐Rhap‐(1⟶, ⟶3)‐Araf‐(1⟶, GalpA‐(1⟶, ⟶5)‐Araf‐(1⟶, ⟶2,3)‐Rhap‐(1⟶, ⟶2,4)‐Rhap‐(1⟶, ⟶3)‐Galp‐(1⟶, ⟶4)‐Galp‐(1⟶, ⟶4)‐GalpA‐(1⟶, ⟶4)‐Glcp‐(1⟶, ⟶2,5)‐Araf‐(1⟶, ⟶3,4)‐Galp‐(1⟶, and ⟶2,4)‐Glcp‐(1⟶, in a molar ratio of 1.75:5.81:5.55:1.95:1.98:2.76:8.90:8.77:1.44:3.35:1.76:8.49:33.88:1.66:2.27:5.44:4.25. These conclusions indicate that the relatively complex structure of UEDAP‐4 can be attributed to its composition of 17 distinct sugar residues.

**TABLE 4:** Glycosidic linkage composition of methylated UEDAP‐4.

Retention time (min)	Methylated sugar	Linkage type	Characteristic ions (m/z)	Molar ratio (%)
5.592	2,3,4‐Me_3_‐Rhap	Rhap‐(1⟶	59, 72, 89, 102, 115, 118, 131, 145, 162, 175	1.75
5.907	2,3,5‐Me_3_‐Araf	Araf‐(1⟶	71, 87, 102, 118, 129, 145, 161	5.81
8.556	3,4‐Me_2_‐Rhap	⟶2)‐Rhap‐(1⟶	89, 100, 115, 130, 131, 175, 190	5.55
8.692	2,4‐Me_2_‐Rhap	⟶3)‐Rhap‐(1⟶	87, 89, 101, 118, 131, 234	1.95
8.728	2,3‐Me_2_‐Rhap	⟶4)‐Rhap‐(1⟶	101, 102, 118, 131, 143, 162	1.98
9.245	2,5‐Me_2_‐Araf	⟶3)‐Araf‐(1⟶	87, 99, 113, 118, 129, 201, 233	2.76
9.636	2,3,4,6‐Me_4_‐GalpA	GalpA‐(1⟶	73, 89, 102, 118, 147, 162, 163, 207	8.90
10.404	2,3‐Me_2_‐Araf	⟶5)‐Araf‐(1⟶	87, 102, 118, 129, 162, 189	8.77
11.100	4‐Me‐Rhap	⟶2,3)‐Rhap‐(1⟶	71, 89, 101, 131, 262	1.44
12.475	2,4,6‐Me_3_‐Galp	⟶3)‐Galp‐(1⟶	87, 101, 118, 129, 161, 202, 234	1.76
13.430	2,3,6‐Me_3_‐Galp	⟶4)‐Galp‐(1⟶	87, 102, 113, 118, 129, 162, 233	8.49
13.433	2,3,6‐Me_3_‐GalpA	⟶4)‐GalpA‐(1⟶	87, 99, 102, 115, 118, 131, 162, 175, 235	33.88
13.756	2,3,6‐Me_3_‐Glcp	⟶4)‐Glcp‐(1⟶	87, 102, 113, 118, 129, 162, 233	1.66
14.054	3‐Me‐Araf	⟶2,5)‐Araf‐(1⟶	87, 88, 129, 130, 189, 190	2.27
15.382	2,6‐Me_2_‐Galp	⟶3,4)‐Galp‐(1⟶	87, 118, 131, 143, 185, 205, 307	5.44
16.577	3,6‐Me_2_‐Glcp	⟶2,4)‐Glcp‐(1⟶	87, 88, 99, 113, 130, 190, 233	4.25

#### 3.4.5. NMR Spectroscopy Analysis

The structure of UEDAP‐4 was elucidated using NMR spectroscopy. For clarity in subsequent analysis, the following glycosidic linkages were designated as A to Q: Rhap‐(1⟶ (A), Araf‐(1⟶ (B), ⟶2)‐Rhap‐(1⟶ (C), ⟶3)‐Rhap‐(1⟶ (D), ⟶4)‐Rhap‐(1⟶ (E), ⟶3)‐Araf‐(1⟶ (F), GalpA‐(1⟶ (G), ⟶5)‐Araf‐(1⟶ (H), ⟶2,3)‐Rhap‐(1⟶ (I), ⟶2,4)‐Rhap‐(1⟶ (J), ⟶3)‐Galp‐(1⟶ (K), ⟶4)‐Galp‐(1⟶ (L), ⟶4)‐GalpA‐(1⟶ (M), ⟶4)‐Glcp‐(1⟶ (N), ⟶2,5)‐Araf‐(1⟶ (O), ⟶3,4)‐Galp‐(1⟶ (P), and ⟶2,4)‐Glcp‐(1⟶ (Q).

As shown in Figure 8a, the ^1^H NMR spectrum revealed the following anomeric proton chemical shifts: 5.04 ppm (A, H, C, I), 5.10 ppm (D, K), 4.92 ppm (N), 4.71 ppm (E), 5.06 ppm (B, G, F, M, J), 4.56 ppm (L), 5.14 ppm (O), 4.66 ppm (P), and 5.31 ppm (Q). In the ^13^C NMR spectrum (Figure 8b), the anomeric carbon chemical shifts were observed at 102.89, 107.84, 99.38, 102.44, 102.86, 107.48, 99.35, 107.84, 103.24, 99.44, 103.20, 106.73, 99.41, 102.44, 107.36, 104.94, and 99.45 ppm. These chemical shifts correspond to the anomeric carbons of sugar residues A through Q. These results indicate that UEDAP‐4 consists of arabinose, galactose, rhamnose, glucose, and galacturonic acid residues, consistent with the methylation analysis results. Typically, the anomeric carbon chemical shifts of arabinose residues are greater than *δ*105 ppm, corresponding to α‐configuration glycosidic bonds. Conversely, chemical shifts below *δ*105 ppm correspond to β‐configuration. Therefore, B, F, H, and O are identified as α‐configuration arabinose residues (Cardoso et al., 2002). For rhamnose residues, anomeric proton chemical shifts above *δ*5.0 ppm indicate α‐configuration glycosidic bonds, while shifts below *δ*5.0 ppm indicate β‐configuration. Residues A, C, D, I, and J were identified as α‐configuration rhamnose residues (Lee et al., 2010; Yao et al., 2021; Zhang X. et al., 2013). The C‐5 chemical shift of E (below *δ*72 ppm) confirmed its α‐configuration as a rhamnose residue (Vinogradov et al., 2005). For galactose and galacturonic acid residues, anomeric carbon chemical shifts below *δ*100 ppm correspond to α‐configuration glycosidic bonds. In contrast, shifts above *δ*100 ppm indicate β‐configuration. Therefore, M and G are α‐configuration galacturonic acid residues, while K, L, and P are β‐configuration galactose residues (Xu M. J. et al., 2020). For glucose residues, anomeric carbon chemical shifts below *δ*101 ppm correspond to α‐configuration glycosidic bonds. In contrast, shifts above *δ*101 ppm correspond to β‐configuration glycosidic bonds. Therefore, Q is an α‐configuration glucose residue, while N is a β‐configuration glucose residue (Wang H. X. et al., 2015).

FIGURE 8The NMR spectra of UEDAP‐4 in D_2_O. (a) ^1^H NMR, (b) ^13^C NMR, (c) DEPT‐135, (d) ^1^H‐^13^C HSQC, (e) ^1^H‐^13^C HMBC, (f) ^1^H‐^1^H NOESY, and (g) ^1^H‐^1^H COSY.

**Figure:**
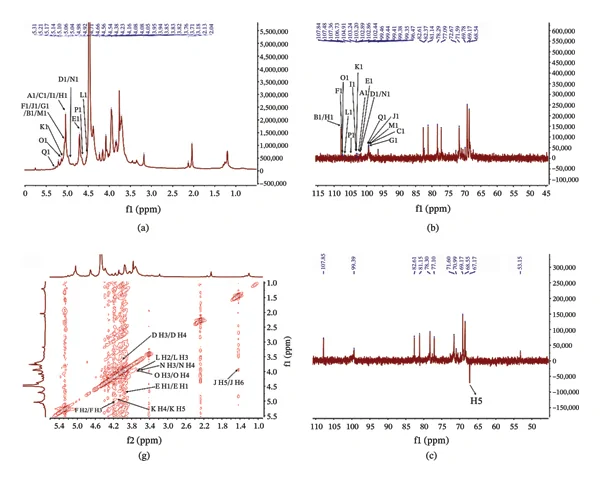

**Figure:**
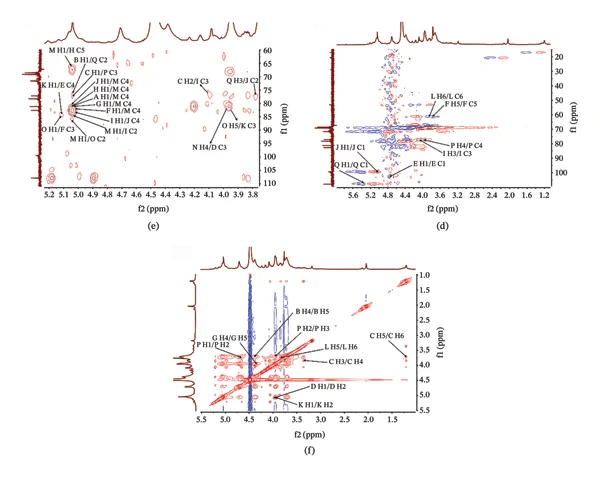

The chemical shifts of H‐1 to H‐6 for each residue were assigned using the adjacent proton correlations observed in the ^1^H–^1^H COSY and ^1^H–^1^H NOESY spectra (Figure 8f,g). In the heteronuclear region of the ^1^H‐^13^C HSQC spectrum, the following H/C correlation peaks were observed: 5.06/99.44 ppm, 5.31/99.46 ppm, 4.71/102.86 ppm, 4.10/77.10 ppm, 4.38/82.56 ppm, 3.73/61.55 ppm, and 3.85/61.24 ppm (Figure 8d). Based on these analyses, the complete ^1^H and ^13^C NMR chemical shift assignments are summarized in Table 5. In the ^1^H‐^13^C HMBC spectrum (Figure 8e), several inter‐residue cross‐peaks were observed: A H‐1/M C‐4 (5.04/80.16 ppm), G H‐1/M C‐4 (5.06/80.16 ppm), M H‐1/H C‐5 (5.06/67.26 ppm), H H‐1/M C‐4 (5.04/80.16 ppm), M H‐1/O C‐2 (5.06/86.43 ppm), O H‐5/K C‐3 (3.95/81.23 ppm), O H‐1/F C‐3 (5.14/84.17 ppm), K H‐1/E C‐4 (5.10/83.34 ppm), F H‐1/M C‐4 (5.06/80.16 ppm), M H‐1/I C‐2 (5.06/84.13 ppm), C H‐2/I C‐3 (4.09/77.10 ppm), I H‐1/J C‐4 (5.04/81.90 ppm), C H‐1/P C‐3 (5.04/77.10 ppm), N H‐4/D C‐3 (3.95/81.53 ppm), J H‐1/M C‐4 (5.06/80.16 ppm), B H‐1/Q C‐2 (5.06/77.89 ppm), and Q H‐3/J C‐2 (3.78/77.10 ppm). The cross‐peaks between residues A H‐1 and M C‐4 indicate that the α‐L‐Rhap‐(1⟶ of H‐1 at *δ*5.03 ppm exhibits an HMBC‐related signal with ⟶4)‐α‐D‐GalpA‐(1⟶ of C‐4 at *δ*80.16 ppm. This observation suggests that α‐L‐Rhap‐(1⟶ is linked to ⟶4)‐α‐D‐GalpA‐(1⟶ at C‐4. The cross‐peaks G H‐1 and M C‐4 indicate that α‐D‐GalpA‐(1⟶ of H‐1 at *δ*5.05 ppm and ⟶4)‐α‐D‐GalpA‐(1⟶ of C‐4 at *δ*80.16 ppm exhibit HMBC‐correlated signals, suggesting that α‐D‐GalpA‐(1⟶ is linked to ⟶4)‐α‐D‐GalpA‐(1⟶ of C‐4. The cross‐peaks M H‐1 and H C‐5 demonstrate that ⟶4)‐α‐D‐GalpA‐(1⟶ of H‐1 at *δ*5.10 ppm and ⟶5)‐α‐L‐Araf‐(1⟶ of C‐5 at *δ*67.26 ppm show HMBC‐related signals, indicating that ⟶4)‐α‐D‐GalpA‐(1⟶ is that the signal at *δ*3.95 ppm for ⟶2,5)‐α‐L‐Araf‐(1⟶ exhibits an HMBC correlation with the signal at *δ*81.23 ppm for ⟶3)‐β‐D‐Galp‐(1⟶ of C‐3. This finding suggests that ⟶2,5)‐α‐L‐Araf‐(1⟶ is linked to ⟶3)‐β‐D‐Galp‐(1⟶ at C‐3. Additionally, the cross‐peaks O H‐1 and F C‐3 indicate that the signal at *δ*5.14 ppm for ⟶2,5)‐α‐L‐Araf‐(1⟶ correlates with the signal at *δ*84.17 ppm for ⟶3)‐α‐L‐Araf‐(1⟶ of C‐3 in an HMBC analysis. This observation implies that ⟶2,5)‐α‐L‐Araf‐(1⟶ is connected to ⟶3)‐α‐L‐Araf‐(1⟶ at C‐3. The remaining cross‐peaks follow a similar pattern. The final structure of UEDAP‐4, illustrated in Figure 9, consists of a main chain: α‐D‐GalpA‐(1 ⟶ 5)‐α‐L‐Araf‐(1 ⟶ 4)‐α‐D‐ GalpA‐(1 ⟶ 2)‐α‐L‐Araf‐(1 ⟶ 3)‐α‐L‐Araf‐(1 ⟶ 4)‐α‐D‐GalpA‐(1 ⟶ 2)‐α‐L‐Rhap‐(1 ⟶ 4)‐α‐L‐Rhap‐(1 ⟶ 4)‐α‐D‐GalpA‐(1⟶, with one terminus at R1 and branched chains at R2, R3, and R4.

**TABLE 5:** Chemical shift of ^1^H and ^13^C NMR in UEDAP‐4.

Residues	Linkage		1	2	3	4	5	6
A	Rhap‐(1⟶	C	102.89	71.6	71.25	72.61	70.40	17.04
		H	5.04	4.25	4.33	3.42	3.84	1.40

B	Araf‐(1⟶	C	107.84	82.6	77.1	84.3	61.55	
		H	5.06	4.08	3.86	4.36	3.73	

C	⟶2)‐Rhap‐(1⟶	C	99.38	82.35	68.55	70.27	68.77	17.04
		H	5.04	4.09	3.83	3.36	3.73	1.21

D	⟶3)‐Rhap‐(1⟶	C	102.44	68.80	81.53	70.60	70.69	17.04
		H	5.10	4.20	4.00	3.56	3.35	1.21

E	⟶4)‐Rhap‐(1⟶	C	102.86	69.18	68.55	83.34	69.35	17.04
		H	4.71	3.95	3.83	3.34	4.05	1.21

F	⟶3)‐Araf‐(1⟶	C	107.48	81.15	84.17	77.10	61.55	
		H	5.06	4.20	3.96	3.86	3.73	

G	GalpA‐(1⟶	C	99.35	70.40	70.15	69.18	71.25	173.82
		H	5.06	3.51	3.72	3.94	4.33	

H	⟶5)‐Araf‐(1⟶	C	107.84	81.9	77.10	82.60	67.26	
		H	5.04	4.09	3.95	4.08	3.93	

I	⟶2,3)‐Rhap‐(1⟶	C	103.24	84.13	77.1	72.67	69.22	17.04
		H	5.04	4.36	4.10	3.47	3.80	1.31

J	⟶2,4)‐Rhap‐(1⟶	C	99.44	77.10	69.35	81.90	69.18	17.04
		H	5.06	4.00	3.82	4.09	3.96	1.40

K	⟶3)‐Galp‐(1⟶	C	103.20	75.15	81.23	68.73	77.10	61.20
		H	5.10	3.96	3.67	4.20	3.95	3.68

L	⟶4)‐Galp‐(1⟶	C	106.73	71.35	74.98	79.67	75.21	61.24
		H	4.56	3.63	3.96	4.16	3.77	3.85

M	⟶4)‐GalpA‐(1⟶	C	99.41	68.55	69.18	80.16	71.60	171.17
		H	5.06	3.83	3.94	4.40	4.71	

N	⟶4)‐Glcp‐(1⟶	C	102.44	72.67	77.23	82.44	77.10	61.26
		H	4.92	3.42	3.68	3.95	3.72	3.89

O	⟶2,5)‐Araf‐(1⟶	C	107.36	86.43	81.01	84.38	69.18	
		H	5.14	4.20	3.98	3.68	3.95	

P	⟶3,4)‐Galp‐(1⟶	C	104.91	71.03	77.11	82.56	75.16	61.24
		H	4.66	3.71	3.95	4.38	3.72	3.85

Q	⟶2,4)‐Glcp‐(1⟶	C	99.46	77.89	77.22	78.58	70.4	61.16
		H	5.31	3.42	3.78	3.46	3.51	3.85

FIGURE 9(a) Predictive structural formula for UEDAP‐4 and (b) predictive model diagram for UEDAP‐4.

**Figure:**
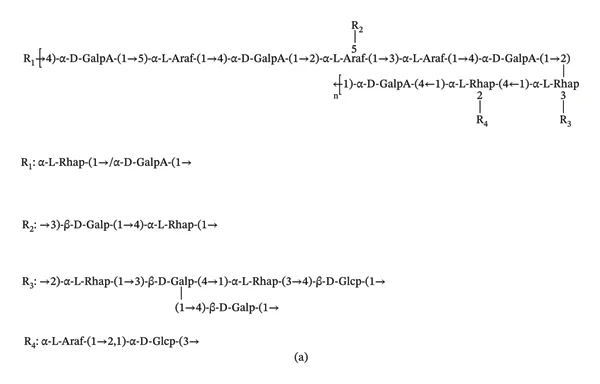

**Figure:**
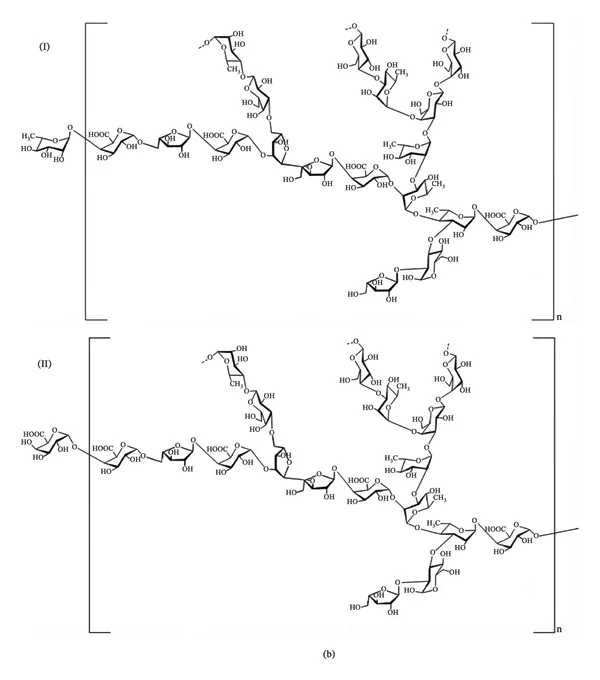

### 3.5. Antioxidant Activity Assay

**FIGURE 10:**
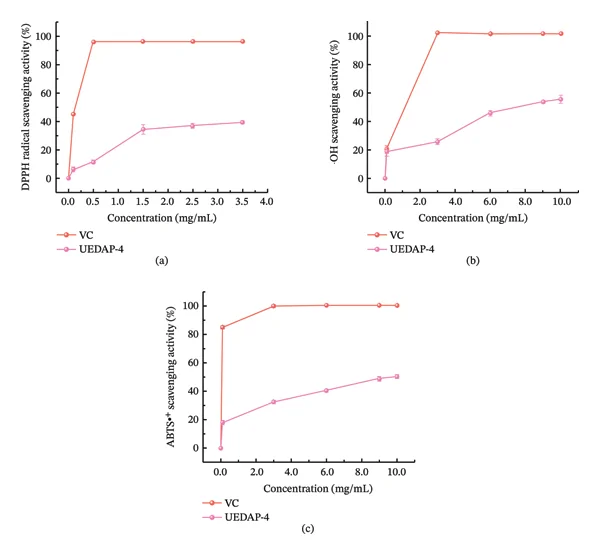
Scavenging activity of UEDAP‐4 and Vc on free radicals: (a) DPPH free radicals, (b) hydroxyl free radicals, and (c) ABTS free radicals.

As shown in Figure 10a, it can be observed that the higher the mass concentration of UEDAP‐4 and Vc, the greater their ability to scavenge DPPH·. When the mass concentration of UEDAP‐4 is 3.5 mg/mL, its scavenging rate of DPPH· reaches its maximum value of 39.41 ± 0.96%. The IC_50_ value of UEDAP‐4 for DPPH· was determined to be 5.399 mg/mL. Although UEDAP‐4 demonstrated a lower DPPH· scavenging capacity than Vc at equivalent concentrations, its ability to eliminate DPPH· remained significant. As illustrated in Figure 10b, the scavenging effect of ·OH increased with the rise in mass concentration of UEDAP and Vc. The ·OH scavenging activity of UEDAP‐4 increased significantly across the concentration range of 0.0–9.0 mg/mL. At a concentration of 10.0 mg/mL, UEDAP‐4 reached its maximum ·OH scavenging activity of 55.65 ± 2.68%. The IC_50_ value of UEDAP‐4 for ·OH was calculated as 8.984 mg/mL, confirming its notable scavenging ability, although it remained lower than that of Vc at equivalent concentrations. As demonstrated in Figure 10c, the ABTS^⋅+^ scavenging capacity of UEDAP‐4 and Vc increased with higher mass concentrations. At a mass concentration of 10.0 mg/mL, UEDAP‐4 achieved its maximum ABTS^⋅+^ scavenging rate of 50.43 ± 1.40%. The IC50 value of UEDAP‐4 for ABTS^⋅+^ was determined to be 14.151 mg/mL, demonstrating its significant scavenging capacity at this concentration. However, UEDAP‐4 exhibited a lower ABTS^⋅+^ scavenging capacity compared to Vc at equivalent concentrations. Previous studies have demonstrated that polysaccharides with higher contents of GalA, Ara, Rib, Man, and Glc exhibit enhanced antioxidant activity, whereas increased levels of Fru and Gal are associated with reduced antioxidant capacity [43, 44]. Therefore, the superior scavenging capacity of UEDAP‐4 for DPPH·, ·OH, and ABTS·^+^ can be attributed to its elevated levels of GalA and Ara.

## 4. Conclusion

In this study, polysaccharides were extracted from Xinjiang dried apricots using ultrasound‐assisted enzymatic extraction. The optimal extraction conditions for polysaccharides were determined using a combination of one‐way tests and response surface methodology. The ideal parameters identified for the extraction process are as follows: a liquid‐to‐feed ratio of 30 mL/g, an extraction time of 25 min, an extraction temperature of 50°C, a composite enzyme ratio of 2.0, a concentration of composite enzyme at 2.0%, and a pH level of 5.5. Under these optimized conditions, the polysaccharide yield reached 35.53 ± 0.60 g per 100 g of dried apricot powder (35.53 ± 0.60% w/w).

Crude polysaccharides from dried apricots were separated and purified by DEAE‐cellulose DE‐52 anion exchange column chromatography, yielding UEDAP‐4 as the isolated fraction. The structural characteristics of UEDAP‐4 were determined using HPGPC, GC–MS, FTIR, and NMR analyses. UEDAP‐4 was identified as a homogeneous polysaccharide consisting of five monosaccharides: Ara, Rha, Gal, Glc, and GalA. The molar ratios of these monosaccharides were determined to be 28.78:20.36:6.87:5.51:38.48. The main chain of UEDAP‐4 comprises the following repeating units: ⟶4)‐α‐D‐GalpA‐(1 ⟶ 5)‐α‐L‐ Araf‐(1 ⟶ 4)‐α‐D‐GalpA‐(1 ⟶ 2)‐α‐L‐Araf‐(1 ⟶ 3)‐α‐L‐Araf‐(1 ⟶ 4)‐α‐D‐GalpA‐(1 ⟶ 2)‐α‐L‐Rhap‐(1 ⟶ 4)‐α‐L‐Rhap‐(1 ⟶ 4)‐α‐D‐GalpA‐(1⟶. Additionally, the structure features three branched chains attached to C5 of ⟶2,5)‐α‐L‐Araf‐(1⟶, C3 of ⟶2,3)‐α‐L‐Rhap‐(1⟶, and C2 of ⟶2,4)‐α‐L‐Rhap‐(1⟶. Furthermore, UEDAP‐4 demonstrated significantly enhanced scavenging activity against DPPH·, ·OH, and ABTS^·+^ radicals. This enhanced antioxidant activity can be primarily attributed to the high content of GalA and Ara in UEDAP‐4.

Therefore, a deeper understanding of the relationship between the structural characteristics of acidic UEDAP‐4 and its in vitro free radical scavenging efficacy could facilitate its potential applications in diverse fields, such as the food industry, healthcare, and cosmetics. However, further research is necessary to elucidate the underlying mechanisms of its structure–activity relationship and address unresolved questions in future studies.

## Author Contributions

Hongying Ma: writing–original draft, methodology, investigation, formal analysis, and data curation. Xiaotian Wang: investigation and formal analysis. Zhongyang Li: writing–review and editing and conceptualization. Huakun Ying: methodology and conceptualization. Cheng Ma: formal analysis and conceptualization. Shuailian Zhao: investigation and data curation. Jiangli Lin: writing–review and editing, resources, project administration, investigation, and funding acquisition. Bin Wu: supervision, methodology, and investigation.

## Funding

This work was supported by the Science and Technology Department of Xinjiang Uygur Autonomous Region (2022B03006‐1).

## Conflicts of Interest

The authors declare no conflicts of interest.

## Data Availability

The data that support the findings of this study are available on request from the corresponding author. The data are not publicly available due to privacy or ethical restrictions.
